# AgBioDatabase Finder: an online tool to help researchers find and submit agricultural genomic, genetic, and breeding data

**DOI:** 10.17912/micropub.biology.001896

**Published:** 2025-11-19

**Authors:** Leyla Cabugos, Katheryn Buble, Jenna Daenzer, Sook Jung, Dorrie Main, Annarita Marrano, David Molik, Daniela Raciti, Adam Wright, Karen Yook, Leonore Reiser

**Affiliations:** 1 Robert E. Kennedy Library, California Polytechnic State University, San Luis Obispo, California, United States; 2 Department of Horticulture, Washington State University, Pullman, Washington, United States; 3 Genetics Society of America, Rockville, Maryland, United States; 4 Phoenix Bioinformatics, Newark, California, United States; 5 Center for Scholarly Publishing, Kansas State Libraries, Kansas State University, Manhattan, Kansas, United States; 6 Division of Biology and Biological Engineering, California Institute of Technology, Pasadena, California, United States; 7 Adaptive Oncology Department, Ontario Institute for Cancer Research, Toronto, Ontario, Canada

## Abstract

To improve the FAIRness of agricultural genomic, genetic, and breeding ( GGB ) data, the AgBioData FAIR Scientific Literature Working Group developed a free search tool that helps researchers identify appropriate databases for submitting their data. Existing repository discovery tools lack the specificity needed for GGB data. Our tool filters databases by organism and data type, mapped using established ontologies, and provides database profiles linking directly to submission guidelines. By guiding authors toward suitable, domain-specific databases, this resource facilitates improved data curation, discoverability, and reuse in agricultural research. The tool can be accessed at https://www.agbiodata.org/databasefinder.

**Figure 1. AgBioDatabase Finder Tool:**
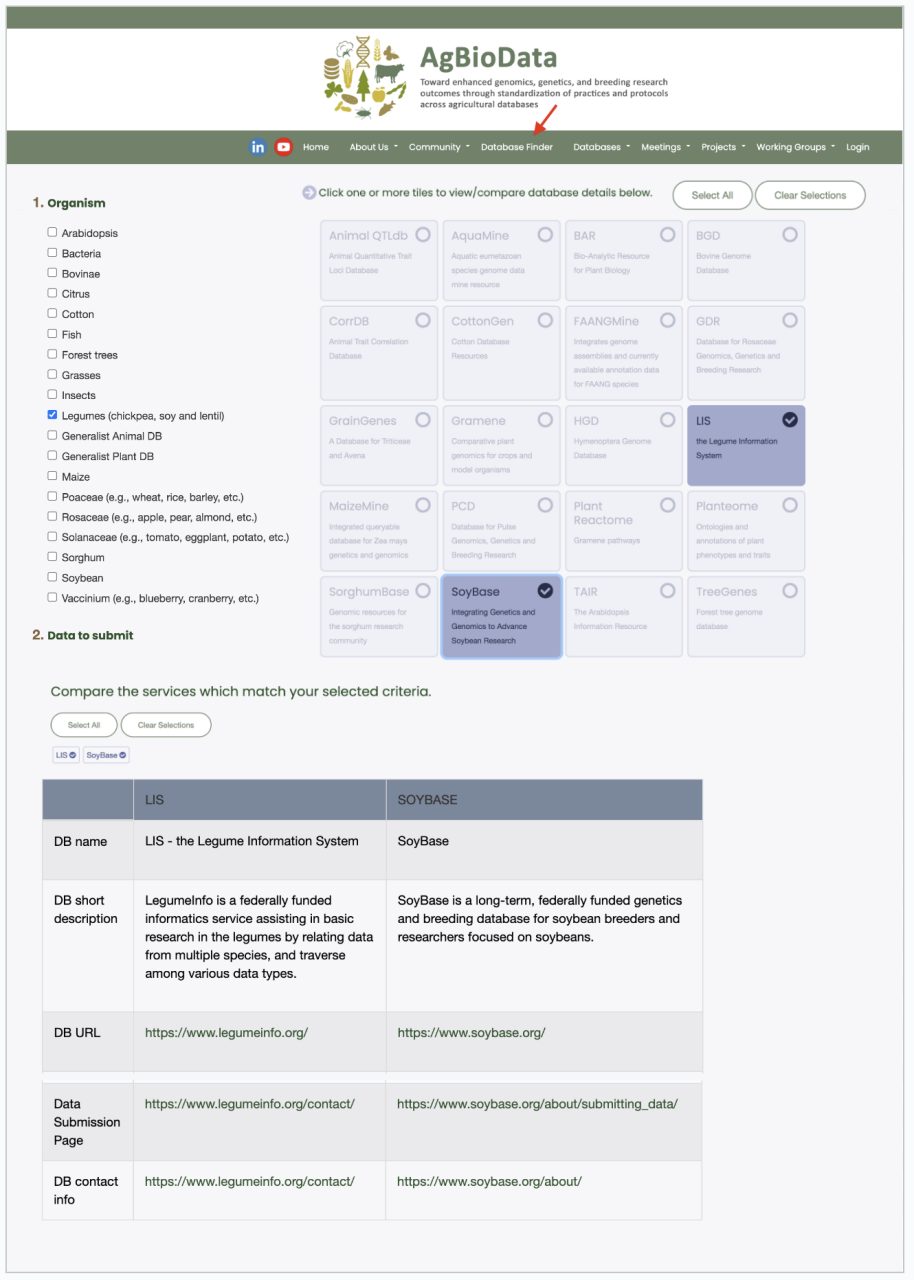
Representative image of the tool showing its location on the AgBioData website, and expanded profiles of two databases, selected for comparison, which meet filter criteria indicated at left.

## Description

AgBioData is a consortium of 45 agricultural biological databases and related resources convened with the mission of consolidating standards and best practices for genomic, genetic, and breeding (GGB) data management ( https://www.agbiodata.org/ ; Harper et al., 2018). Members of the consortium collaborate through a variety of working groups, each with specific objectives. The AgBioData FAIR Scientific Literature Working Group, established in 2023, aims to identify and address bottlenecks in the integration of research data into public databases to enhance their discoverability and reusability. Curators for AgBioData member databases routinely search the published literature for curatable data, though ideally such data would be submitted directly to their databases by the authors. Direct data submission processes for specialty databases guide authors in preparing and describing their data, reducing the labor-intensive task of extracting and standardizing data after publication and without author input. This also prevents papers from being published that lack critical metadata and/or have data that were not properly deposited in public databases.

Journal data sharing policies provide an important incentive for authors to share the data associated with a research publication. Many journals guide data deposition for some common data types, such as sequences or gene expression, into primary databases, but lack guidance for data for which community or model organism databases are the optimal venue. These types of databases typically focus on curation of a single species or taxon and often host data that are not curated anywhere else. Thus, the Working Group sought to locate or develop a tool to help authors and journal editors identify the most appropriate data repository for their publications, ensuring that the reported data are not only open but also FAIR, that is, Findable, Accessible, Interoperable and Reusable (Wilkinson et al., 2016).

The Working Group examined existing guidance on where to submit agricultural data (specifically, QTL maps, QTL markers, variant and phenotype data), by surveying available resources and reviewing recommendations provided in journal author guidelines. Two key free resources emerged: FAIRsharing (FAIRsharing.org), a discovery tool that catalogs over 2,300 databases, standards, and policies (the FAIRSharing Community, et al., 2019); and re3data (re3data.org; Pampel et al., 2013), which indexes global research repositories that span all disciplines from life sciences and engineering to social sciences. While these free tools provide high-level guidance to authors across many scientific disciplines and can effectively aid database discovery, their purpose as designed is to be complete catalogs, databases, schemas, and policies. Consequently, these tools are often not optimized for the biocurator's goal of highest impact return for submission. The AgBioDatabase Finder tool draws from (and complements) complete catalog tools, like FAIRsharing, to support author decision-making.

Advanced artificial intelligence tools can help researchers answer the question of where to submit their data and in what format. Dataseer is a machine-learning-driven service that identifies shareable datasets within manuscripts, recommends appropriate repositories, and offers guidance on data preparation (Marill, 2020; dataseer.ai ). However, it is primarily geared towards funders, institutions, and publishers to help them streamline checking for compliance with open data policies. We perceived an unmet need for an accessible (free) tool to guide individual researchers on where to deposit GGB data. To address these challenges, we developed AgBioDatabase Finder, a granular tool that complements and could eventually be integrated with established general-purpose resources such as FAIRsharing.

During the development process, we also considered whether researchers can use popular virtual assistants driven by Large Language Models (LLM) to generate useful recommendations of where to deposit their data. When we began to manually aggregate information on our member databases, we noted that their web portals do not always clearly identify what type of data they accept. If this is not obvious to us, we reason that it would be extremely difficult for an LLM like ChatGPT to make a recommendation. Our effort to aggregate information about specialist databases (which culminated in the dataset that underlies this tool, see Extended Data), particularly allowed us to work with AgBioData members to generate standardized and detailed database profiles, including descriptions of entities, properties, and relationships, which may facilitate more accurate recommendations by LLMs.

Building on this standardized database information, AgBioDatabase Finder is a database locator tool for agricultural genomic, genetic, and breeding data that allows users to quickly identify databases that are optimized for their study organism(s) and datatype(s) to make the data as FAIR as possible (Figure 1). The default display presents brief profiles of all included databases. We chose to offer three options to filter this list: “Organism” (using the categories offered by database managers in populating their profiles, which vary in granularity), “Data to Submit” (data types accepted via author submission), and “Data to Find” (data hosted in a database, including via non-author initiated processes). The latter option also enables the use of the tool for data discovery. The data types are represented using ontology terms mapped to data types collected by the AgBioData Genotype to Phenotype Working Group (Deng et al. 2023). We primarily used the EDAM ontology (Black et al., 2022) of common concepts in the life sciences and associated disciplines to describe data types, supplementing it with other ontologies (as indicated in the Datatype definitions and source ontologies tab of the Extended Data file) when suitable terms were unavailable.

When an organism or data type is selected from the list of filter options, database profiles that do not match these criteria are grayed out but remain visible. This approach highlights the relevant options while avoiding blank search results by clearly showing which databases match the selected criteria. Filter options are combined using an AND operator, as the program template does not support complex logic. Clicking on a short profile reveals more detailed information, including a list of accepted data types and links to data submission pages. Users can select multiple profiles to display their detailed information side-by-side. An example of the database comparison is shown in Figure 1.

AgBioDatabase Finder represents an important contribution to the agricultural research community, offering a specialized and user-friendly solution for identifying relevant community databases that offer a higher level of data curation for their specialized datasets and the most suitable repositories for agricultural GGB data. This tool can also be used by publishers and generalist repositories to guide authors and data generators on the best places to deposit their data. Ultimately, AgBioDatabase Finder is more than just a tool for finding databases—it is an essential part of the larger effort to ensure that agricultural data are properly curated, accessible, and reusable at the time of publication. By empowering researchers to make informed decisions about where to deposit their data, we aim to contribute to a more robust, interoperable, and sustainable agricultural data ecosystem.

## Methods

We identified a workable template for our database finder tool in an open-source program developed in Drupal (currently in version 10) for Cornell University's Data Storage Finder (Cornell University Research Data Management Service Group and Cornell Information Technologies Custom Development Group, 2018), which was designed to help Cornell-affiliated authors evaluate data storage options.

We gathered input from representatives of AgBioData membership on the utility and intended features of the tool at an annual meeting and via a short follow-up survey. Three representatives of AgBioData member databases outside the working group tested and provided feedback on the prototype.

At present, the tool must be populated manually by moderators. We circulated a form to gather information from which to create profiles for all AgBioData member databases that are in scope for the Finder tool.&nbsp; As new members join the consortium, we will work with their representatives to add their profile. Asking member database managers to identify data submission instructions on their websites encouraged some to update and improve this guidance.

**Data Sources**

The repositories currently presented in this tool are members of the AgBioData Consortium. While the tool's primary purpose is to help authors find suitable repositories for sharing their data, we also included a few of the member databases that do not accept direct author submissions to facilitate data discovery. In the future we plan to include information and recommendations on public data repositories that AgBioData member databases draw from, such as European Nucleotide Archive, ArrayExpress, and GEO.

## Data Availability

Description: Source Data and Ontology Terms for AgBioDatabase Finder Profiles. The tab ‘AgBioDatabase Source data’ contains the descriptive information used to populate the database profiles currently present in the AgBioDabase Finder. The tab ‘Datatype definitions and source ontologies’ contains a list of terms for data types supported by the tool, a link to the source and definition of the ontology term, and a streamlined definition used in the AgBioDatabase Finder tool. . Resource Type: Dataset. DOI: https://doi.org/10.22002/11wg8-jgx73
